# Identification of individual subjects on the basis of their brain anatomical features

**DOI:** 10.1038/s41598-018-23696-6

**Published:** 2018-04-04

**Authors:** Seyed Abolfazl Valizadeh, Franziskus Liem, Susan Mérillat, Jürgen Hänggi, Lutz Jäncke

**Affiliations:** 1 Division Neuropsychology, Department of Psychology, University of Zurich, Zurich, Switzerland; 2 Sensory-Motor Systems Lab, Department of Health Sciences and Technology, ETH Zurich, Zurich, Switzerland; 3 International Normal Aging and Plasticity Imaging Center (INAPIC), University of Zurich, Zurich, Switzerland; 4 University Research Priority Program (URPP) “Dynamics of Healthy Aging”, University of Zurich, Zurich, Switzerland

## Abstract

We examined whether it is possible to identify individual subjects on the basis of brain anatomical features. For this, we analyzed a dataset comprising 191 subjects who were scanned three times over a period of two years. Based on FreeSurfer routines, we generated three datasets covering 148 anatomical regions (cortical thickness, area, volume). These three datasets were also combined to a dataset containing all of these three measures. In addition, we used a dataset comprising 11 composite anatomical measures for which we used larger brain regions (11LBR). These datasets were subjected to a linear discriminant analysis (LDA) and a weighted K-nearest neighbors approach (WKNN) to identify single subjects. For this, we randomly chose a data subset (training set) with which we calculated the individual identification. The obtained results were applied to the remaining sample (test data). In general, we obtained excellent identification results (reasonably good results were obtained for 11LBR using WKNN). Using different data manipulation techniques (adding white Gaussian noise to the test data and changing sample sizes) still revealed very good identification results, particularly for the LDA technique. Interestingly, using the small 11LBR dataset also revealed very good results indicating that the human brain is highly individual.

## Introduction

Is it possible to identify an individual subject on the basis of particular brain anatomical features provided by the frequently used and widely available Freesurfer tool? This question has not been answered nor studied so far. However, anyone who is working with MRI-based reconstructions of the human brain is likely to have the impression that human brains are highly individual. This individuality seems to be obvious when inspecting shape and size of the gyri and sulci, or the volume and form of the entire brain.

Although the individuality of the human brain has not been demonstrated scientifically so far, there are many scientific reports showing that many features of the human brain are modulated by genetic^1^, non-genetic biological^2^, and environmental^3–5^ influences interacting in a currently unknown manner. If brain anatomy depends on these individual influences, it should be possible to identify individual subjects on the basis of neuroanatomical and neurophysiological features using sophisticated statistical procedures. In this paper, we will demonstrate that it is indeed possible to identify individual subjects on the basis of specific neuroanatomical features by applying mathematical classifications and identification tools. By applying these techniques, we will be able to scientifically substantiate the often-reported impression that specific features of the human brain are individual, providing the opportunity to identify individual subjects on the basis of particular anatomical features. In addition, we will be in the position to identify those brain features which contribute most to individual subject identification.

Several papers have been published in the last decade reporting more or less successful attempts to identify individual subjects on the basis of neuroanatomical and neurophysiological features. In these studies, EEG measures have mostly been used either obtained from resting state^6–8^ or during the processing of cognitive tasks^9^. A few more recent studies have used BOLD fluctuations measured with fMRI during resting state and during the performance of cognitive tasks. In these studies, subject identification was quite successful, supporting the idea of individual connectivity profiles that can be used to distinguish individual subjects^10–12^. However, only two studies have been published so far describing subject identification attempts on the basis of neuroanatomical measures^13,14^.

In these studies, the authors^13,14^ used a large dataset of MRI brain scans from three public brain databases and used a new mathematical algorithm for shape analysis of anatomical data. With this technique, they developed a software pipeline (BrainPrint), with which they have been able to classify nearly all subjects (99.8%) on the basis of the MRI scans. In their second paper, the same authors expanded their approach and did very well in classifying age and sex on the basis of their BrainPrint pipeline^14^. Based on their results, the authors conclude that human brain structures are unique to individuals and can be used for subject identification. Wachinger^14^ have used a new transformation for analyzing the shape of the brain, which is called ShapeDNA. By using this ShapeDNA they are in the position to calculate eigenvalues and eigenfunctions of the Laplace Beltrami operator using a higher-order finite elements method (FEM with Dirichlet or Neumann boundary condition^15^). The authors claim that this algorithm is useful to map the brain shape information to a new space, so if it is possible to identify individuals by using ShapeDNA, the shape of the human brain should be unique in each subject. The findings of these two studies are intriguing and important in many respects. However, it should be emphasized that they achieved their results by using a specifically developed mathematical algorithm to estimate the individual features of the human brain (e.g., a shape analysis). One might speculate whether the very good and astonishingly high accuracy levels for subject identification might be based simply on the fact that they have developed a new and more efficient technique to mathematically describe and define anatomical features.

In this paper, we will examine whether it is possible to identify individual subjects on the basis of neuroanatomical features when using more standard anatomical measures obtained from the widely used FreeSurfer tool. A major question we are trying to answer with our project is which brain measures are necessary for good subject identification. Is it necessary to use all anatomical information (e.g., cortical thickness, volume, and area) from all anatomical regions provided by the FreeSurfer tool? Or are only some composite measures necessary for achieving good or acceptable results?

With this study, we will explicitly answer the following questions: (1) Is it possible to identify individual subjects on the basis of a combination of anatomical measures? (2) Which combination of anatomical measures is most informative in identifying individual subjects? (3) Do different classification techniques (linear discriminant analysis: LDA; weighted k-nearest neighbor: WKNN) substantially differ in terms of their subject identification accuracy? We have chosen these two techniques because of several reasons: First, LDA is frequently used, easy to apply and provides fast computation times. Second, WKNN, on the other hand, provides generally excellent classification and identification results but requires significant computation resources.

## Methods

### Subjects

191 participants (100 male, 91 female) with three measurement time points were included in the present work. They took part in the Longitudinal Healthy Aging Brain (LHAB) database project^15^. MRI measurements were conducted once a year over a period of two years (t1: baseline measurement; t2: 1 year after baseline; t3: 1 year after t2). At the first time point, subjects were between 64 and 85 years old (M = 70.1 years, SD = 4.8 years). Participants were cognitively healthy, right-handed (as confirmed by the Annett Handedness Questionnaire^16^), had no history of neurological or psychiatric disorder, and did not suffer from migraine, diabetes or tinnitus. They gave written informed consent prior to participating in the study. In addition, all methods were carried out in accordance with relevant guidelines and regulations. All experimental protocols were approved by the ethical committee of the canton of Zurich (KEK-ZH-Nr. 2010–0267). The data of this sample has been used in previous publications of our group^17–20^.

### Preprocessing of Anatomical Data

MRI data were acquired with a 3.0 T Philips Ingenia scanner (Philips Medical Systems, Best, The Netherlands). T1-weighted images were recorded with a gradient echo sequence (3D turbo field echo, 160 sagittal slices, slice thickness = 1 mm, in-plane resolution = 1 × 1 mm, FOV = 240 × 240 mm, repetition time = 8.18 ms, echo time = 3.80 ms, flip angle = 8°). FreeSurfer (v5.3) was used to obtain measurements of cortical and subcortical anatomy^21–23^. After completing the standard recon-all pipeline, measurements for cortical thickness, surface area, and volume were extracted for the regions of the Destrieux (aparc.a2009s) parcellation scheme^24^. Subcortical and global volume measurements were also extracted from FreeSurfer’s aseg segmentation^25^. To ensure independence between time points, FreeSurfer’s cross-sectional (rather than longitudinal) analysis stream was used.

For this paper, we estimated mainly the same anatomical measures as used in our previous age prediction paper^17^. In short, we will reiterate the obtained measures. First, we estimated compartmental cortical volumes, thickness, and surface area measures for 148 brain regions using the FreeSurfer (version 5.3) anatomical region of interest (ROI) tool using the aparc.a2009s parcellation scheme^24^. We also estimated subcortical (thalamus, putamen, pallidum, caudatus, hippocampus, amygdala, and accumbens) and total subcortical volume, the volumes of the corpus callosum (CC), cerebrospinal fluid, total white matter hypointensity, brainstem (midbrain, pons, medulla oblongata, and superior cerebellar peduncle), total brain volume, mean global cortical thickness, and total surface area. The subcortical anatomical measures were obtained using the FreeSurfer’s subcortical segmentation tool (see also^18^). In summary, we obtained the following anatomical measures, which were first divided into five basic anatomical feature sets:

1. Large brain regions (LBR) comprising 11 large (global) brain measures: total cortical volume: CV; mean cortical thickness: CT; total cortical surface area: CA; total cortical gray matter volume: CoGM; total cortical white matter volume: CoWM; total cerebellar gray matter volume: CeGM; total cerebellar white matter volume: CeWM; total subcortical volume: SCV; brainstem volume: BV; corpus callosum volume: CC; white matter hypointensities: WMH (11 LBR),
2. the 148 compartmental cortical thickness measures (THICKNESS),
3. the 148 compartmental cortical surface area measures (AREA),
4. the 148 compartmental cortical volume measures (VOLUME), and
5. the combination of the cortical thickness, area, and volume measures (ALL). This dataset also includes additional brain measures (e.g., CC, ICV, volumes of brain stem, cerebellum, basal ganglia, ventricles) totaling 510 anatomical features (the entire list of ROIs is listed in the supplementary material).

### Statistical Methods Used for Subject Identification

In this study, we have used two identification methods: (1) Linear Discriminant Analysis (LDA) and (2) a modified version of the Weighted K-Nearest Neighbor (WKNN). LDA is one of the simplest classification/identification techniques currently available. LDA classifiers aim at finding the best linear combination of predictors in order to optimize the separation between multiple classes. Often the primary goal of an LDA is to project a feature space onto a smaller subspace while maintaining the class-discriminatory information. The nearest neighborhood (1-NN) rule on the other hand identifies the class of unknown data points based on its nearest neighbor data point, the class of which is already known. The K-Nearest Neighbor classifier (KNN) is a variant of the 1-NN procedure^26^. Cover and Hart propose KNN, in which the nearest neighbor is calculated on the basis of the value k that specifies how many nearest neighbors are to be considered to define a class of a sample data point. For improvement of the KNN technique, several methods have been proposed. In this study, we have applied the so-called weighted KNN (WKNN), which adds weight over a distance function. In addition, we also applied the square inverse distance in the context of the WKNN. The entire code has been written in Matlab by one of this paper’s authors (S.A.V). The code has been validated using standard datasets.

### Training and Application of the Techniques

Before presenting our methodological approach, it is necessary to describe the difference between classification and identification. For classification, the number of classes is smaller than the number of subjects. For identification, the number of classes is identical to the number of subjects; thus, we cannot use the classifier techniques in the way that they are used in the classical classification approaches. If we repeat the measurement for each subject M times (for M > 2), we have enough samples for each class (here each subject). In these cases, we can use the above-mentioned classifiers and the results can be used for subject identification. Each subject in the LHAB dataset was scanned three times, with a year in between scans. Therefore, each subject is assumed to be a class with three samples. Two time points were randomly selected for training while the other was used for testing. The time points used for testing and training were selected randomly to ensure no bias on identification. Each class (subject) was evaluated separately. The accuracies of the identifications are reported as accuracy, sensitivity, specificity, and F1-scores. Sensitivity (the true positive rate) reflects the proportion of correctly identified subjects. Specificity on the other hand (the true negative rate) indicates the proportion of negatives that are correctly identified (i.e., here the number of correctly classified cases not belonging to a particular subject). Accuracy represents the proportion of truly classified subjects. The F1-score is the harmonic mean of sensitivity (also called recall) and precision (the proportion of truly classified subject over subjects’ number in the class, also called positive predictive value).

To test whether our identification results depend on the sample size, we conducted our identification calculations for different sample sizes. For this, we randomly chose a particular number of subjects (starting with ten subjects and increasing the sample size by ten). Thus, we obtained 19 samples with different sample sizes (10, 20, 30, 40, 50, … 190 subjects). For each sample, we calculated accuracy, sensitivity, specificity, and the F1-score.

We used a further strategy to test the stability of our identification results, which is analogous to the identification strategy used in many machine learning approaches. For example, when training a neural network to identify faces, the trained network should be able to identify a face from different angles, under different lighting conditions, or when only a fraction of the face is presented^27^. Thus, the classifier should be able to identify the target stimuli (here the face) even when the target quality has been changed or is degraded. To simulate such a condition, we added white Gaussian noise to the anatomical features of the test data. In a first step, we performed our identification tests without any noise. In the next steps, we continuously added noise to all features linearly from 5% to 40%. Thus, we carried out our identification test for nine different noise conditions (no noise, 5%, 10%, 15% … 40%).

For comparison between the identification results obtained by the LDA and WKNN techniques and the different datasets, we first used Cochran’s Q test which is an extension of the McNemar test, when the response variable is dichotomous and there are repeated measures. In case of a significant Cochran’s Q test, we performed subsequent Bonferroni-Holm adjusted McNemar tests using SPSS version 22 for Mac OS X. In total, we performed 12 Cochran’s Q tests. For comparing the performance of the two identification methods, we computed five McNemar tests (one for each dataset). Thus, we computed in total 17 statistical tests, and started the Bonferroni-Holm correction with a p = 0.05/17 = 0.0029^28^. To identify those anatomical measures contributing most to the identification results, we calculated stepwise linear discriminant analysis using. We calculated these stepwise LDAs for the dataset used for training and applied these LDA results to the test dataset.

## Results

### Identification of single subjects

The results of the identification procedures computed for the entire sample are listed in Table 1. As can be observed from this table, the accuracies, sensitivities, specificities, and the F1-scores are consistently high. The identification results are very good and partly perfect. Sensitivity ranges between 0.57 and 1, specificity is always perfect, and F1-scores range between 0.51 and 1.

**Table 1:** Summary of the identification results broken down for the different methods (LDA and WKNN) and the different datasets. Acc: accuracy, Sens: sensitivity, Spec: specificity, F1: F1-score.

	LDA				WKNN			
	Acc	Sens	Spec	F1	Acc	Sens	Spec	F1
All	1.00	0.99	1.00	0.99	1.00	0.70	1.00	0.65
AREA	1.00	0.99	1.00	0.99	1.00	0.99	1.00	0.99
THICKNESS	1.00	0.98	1.00	0.97	1.00	1.00	1.00	1.00
VOLUME	1.00	0.99	1.00	0.99	1.00	1.00	1.00	1.00
11 LBR	1.00	0.92	1.00	0.90	1.00	0.57	1.00	0.51

Table 2 shows the results of the Cochran’s Q tests across the different datasets separately for the two methods. As can be seen from this Table, the identification results do not strongly differ for the different datasets. At the bottom of Table 2, the post hoc McNemar test results are listed. These tests revealed that the identification is better for the datasets ALL, AREA, and VOLUME compared to the ALL dataset.

**Table 2:** Summary of the Cochran’s Q test results for the two identification techniques.

	P-Value	significant McNemar
LDA	***	ALL vs. 11LBR AREA vs. 11LBR VOLUME vs. 11LBR
WKNN	***	ALL vs. AREA ALL vs. THICKNESS All vs. VOLUME ALL vs. 11LBR AREA vs. 11 LBR THICKNESS vs. 11LBR VOLUME vs. 11LBR

***p < 0.001.

Table 3 shows the comparisons between the different methods separately for the different datasets. This analysis revealed that LDA is superior to WKNN for the ALL and 11 LBR datasets. For the other datasets both methods revealed similar results.

**Table 3:** Summary of the McNemar tests comparing the accuracies for the LDA and WKNN techniques broken down for the 5 different datasets.

	Dataset	P value
WKNN vs. LDA	ALL	***
	AREA	n.s.
	THICKNESS	n.s.
	VOLUME	n.s.
	11 LBR	***

***p < *0*.*001*, *s*ignificant after Bonferroni-Holm correction; n.s.: not significant.

### Identification of single subjects using different noise levels

**Figure 1:**
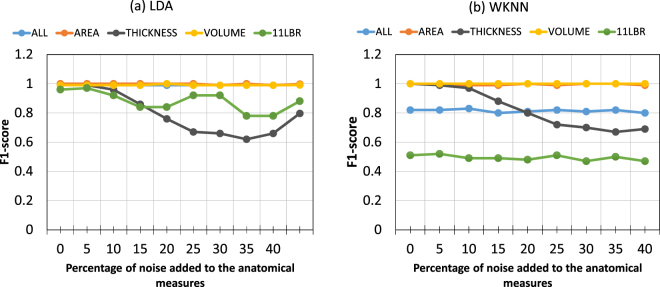
Identification results in the context of different noise levels added to the anatomical measures broken down for the different methods (**a**) LDA and (**b**) WKNN.

Adding noise to the anatomical measures generally results (with few exceptions) in degraded identification ability. Using LDA, we obtained relatively stable identification results for three datasets (ALL, AREA, and VOLUME) across different noise levels (see Fig. 1a). For THICKNESS and 11 LBR, the identification results constantly decreased, but never became smaller than F1 = 0.6. Using the WKNN technique, we obtained stable identification results across different noise levels for the ALL and AREA dataset. A constant low identification result across all noise levels was obtained for the 11 LBR dataset.

Table 4 shows the results of the Cochran’s Q test comparing the identification across the different noise levels separately for each dataset. For the LDA technique, the identification results differ across the different noise levels for the THICKNESS and 11 LBR datasets. For WKNN, we only found a significant noise influence on the identification rate for the THICKNESS dataset

**Table 4:** Summary of the Cochran tests comparing the identification results for the 6 different noise levels separately for the LDA and WKNN techniques.

	LDA	WKNN
ALL	n.s.	n.s.
AREA	n.s.	n.s.
THICKNESS	***^a^	***^c^
VOLUME	n.s.	n.s.
11LBR	***^b^	n.s.

n.s. not significant, ***p < 0.001; ^a–c^: significant differences between 0% noise and all noise additions exceeding 15%.

### Identification of single subjects using different sample sizes

**Figure 2:**
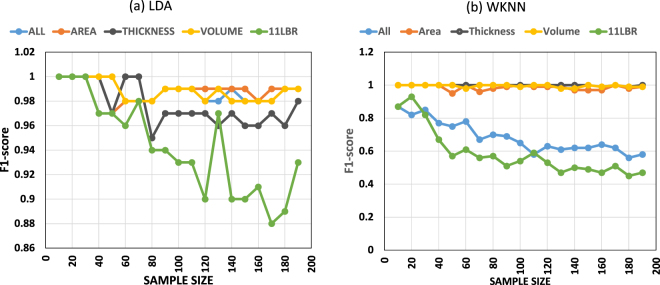
Sample size effect for the different identification techniques and the different datasets. **a**) LDA and **b**) WKNN.

Figure 2a,b indicate the sample size effect for the different identification techniques and the different datasets. As one can observe from this figure, the identification results are very good using the LDA technique even for different sample sizes. The identification results become worse when sample size increases if the WKNN method is used. This happens especially for the 11 LBR and the ALL datasets. For LDA, the results are more or less stable across the different sample sizes. Only the 11 LBR dataset shows a slight decrease in identification accuracy from small to large sample sizes.

### Selection of anatomical features contributing to the subject identification

For highlighting those anatomical features contributing most to subject identification, we conducted stepwise LDAs with the training sample for the ALL and the 11 LBR dataset. To keep the results comparable across the different datasets, we restricted the stepwise regression to 11 variables for both datasets (the ALL dataset containing 510 features while the 11 LBR datasets contains only 11 features). We performed these stepwise LDAs for the randomly selected two time points of measurements (training sample) and applied the results to the data obtained at the other time point (test sample).

The results of the stepwise LDAs and the ALL dataset are shown in Table 5. The identification results for subject identification were better than 91% (F1-value) for the ALL dataset for the test sample. As can be seen from Table 5, several area, volume, and thickness measures from relatively small brain regions are included here. The largest measure included in this list is the left-sided cortex volume.

**Table 5:** Results of the stepwise LDAs for the ALL dataset. Shown are the anatomical measures contributing to the identification result separately for each step.

Step	Feature name	ACC.	SENS.	SPEC.	F1
1	rh_S_oc_middle_and_Lunatus_area	0.99	0.04	0.99	0.03
2	rh_Lat_Fis-ant-Horizont_area	0.99	0.15	1.00	0.12
3	rh_S_interm_prim-Jensen_volume	0.99	0.34	1.00	0.29
4	rh_S_subparietal_area	0.99	0.48	1.00	0.41
5	lh_S_suborbital_thickness	1.00	0.55	1.00	0.50
6	rh_S_oc_middle_and_Lunatus_volume	1.00	0.63	1.00	0.56
7	rh_S_orbital-H_Shaped_area	1.00	0.66	1.00	0.60
8	rh_G_front_middle_area	1.00	0.79	1.00	0.74
9	lhCortexVol	1.00	0.82	1.00	0.78
10	lh_G_temp_sup-Lateral_volume	1.00	0.88	1.00	0.85
11	lh_S_oc_sup_and_transversal_area	1.00	0.93	1.00	0.91

In addition, the identification results (ACC: accuracy, SENS.: sensitivity, SPEC.: specificity, F1: F1 value as a harmonic mean of specificity and sensitivity) are shown for the test sample.

Using the 11 LBR dataset (see Table 6) revealed approximately similar good or even excellent identification results. The brain region contributing most strongly to the subject identification is the total intracranial volume, followed by the total grey matter volume, the volume of cerebrospinal fluid, and the surface of the white matter. With these four measures the F1-value mounts to an F1 = 0.64. Adding all measures of the 11 LBR dataset results in an F1-value of 0.90, with the corpus callosum measure adding no additional information to the 10th step during which mean cortical thickness has been added resulting in an F1-value of F1 = 0.91.

**Table 6:** Results of the stepwise LDAs for the 11 LBR dataset.

Step	Feature name	ACC.	SENS.	SPEC.	F1
1	EstimatedTotalIntraCranialVol	0.99	0.14	1.00	0.11
2	TotalGrayVol	0.99	0.38	1.00	0.32
3	CSF	0.99	0.48	1.00	0.41
4	WhiteSurfArea_area	1.00	0.71	1.00	0.64
5	Cerebellum-Cortex	1.00	0.78	1.00	0.73
6	SubCortGrayVol	1.00	0.90	1.00	0.86
7	CorticalWhiteMatterVol	1.00	0.89	1.00	0.86
8	WM-hypointensities	1.00	0.94	1.00	0.92
9	Cerebellum-White-Matter	1.00	0.89	1.00	0.86
10	MeanThickness_thickness	1.00	0.93	1.00	0.91
11	CC	1.00	0.93	1.00	0.90

Shown are the anatomical measures contributing to the identification result separately for each step. In addition, the identification results (ACC: accuracy, SENS.: sensitivity, SPEC.: specificity, F1: F1 value as a harmonic mean of specificity and sensitivity) are shown for the test sample.

## Discussion

Our study was guided by three study questions: (1) Is it possible to identify individual subjects on the basis of a combination of anatomical measures? (2) Which combination of anatomical measures is most important in identifying individual subjects? (3) Do different classification techniques (linear discriminant analysis: LDA; weighted k-nearest neighbor: WKNN) substantially differ in terms of their subject identification accuracy? With respect to the first study question, we can clearly state that it is indeed possible to identify an individual subject on the basis of their brain anatomical measures. With respect to the second study question, it is obvious that only a few (≈ 11) anatomical features are needed for excellent subject identification. In terms of the third study question, we can say that the relatively simple-to-use LDA method provides good results and is slightly better in terms of subject identification than WKNN. In the following, we will discuss our findings in the context of the current literature and possible applications and future research.

To test whether our identification results remain stable under different conditions, we introduced two data modifications. First, we added white Gaussian noise to the anatomical measures and second, we changed the sample sizes used for training of the classifiers. Adding noise to the anatomical data simulates the often-used strategy in machine learning to examine whether identification still works when the data quality is diminished. This approach is frequently used in machine learning to test whether the used algorithm is efficient to identify features even if the quality of the features is degraded^27^. In our sample, we obtained generally very good identification results even when the “noisy” data were used (see Fig. 1a,b). The relatively simple LDA technique revealed excellent identification results for three datasets (ALL, AREA, and VOLUME) even if 40% Gaussian random noise was added to the anatomical features of the test sample. The identification results are less good for the 11 LBR and THICKNESS datasets. However, the F1 values never dropped below F1 = 0.6, indicating that even moderate sensitivity and specificity is achieved when 40% noise is added to these anatomical measures. For WKNN, we obtained excellent identification results for the VOLUME and AREA datasets across the different noise levels. For the 11 LBR dataset, the results were consistently worse across the different noise levels. A moderate identification result was achieved for the ALL dataset. The identification results are less accurate if the WKNN method was used, particularly for the ALL and 11 LBR datasets. The identification results are fairly similar when using different sample sizes. For LDA, the results are approximately similar across the different sample sizes. For WKNN, the results become worse with increasing sample size for the ALL and 11 LBR datasets.

Taken together, we obtained relatively good (and at times even excellent) identification results using the LDA and WKNN methods even if we modified the samples. However, the relatively easy LDA technique turned out to reveal the best and most stable results across the different datasets and conditions (with noise included). Most interesting is, however, that using the small 11 LBR dataset containing only 11 anatomical composite measures revealed astonishingly good identification results, particularly when using the LDA.

Our study demonstrates that brain anatomical measures are highly individual. Thus, a combination of only a few brain anatomical measures is necessary to identify an individual subject. It is also interesting to note that even the usage of composite anatomical measures representing relatively large brain areas (e.g., total brain volume, total brain area, or mean cortical thickness) are so individual that they can be used for individual subject identification. Our results are interesting because of several issues. First, it again shows that the human brain is an organ with a highly individual architecture driven by specific genes, a specific environment, differential experience, and a combination of all three factors. Thus, our findings complement neuroanatomical research showing that brain anatomical features in humans are related to expertise^29^, experience^30^, learning^31,32^, gender^18^, ethnicity^33,34^, age^35^, giftedness^36^, intelligence^36,37^, personality traits^38^, diagnosed psychiatric diseases^39^, subliminal psychiatric diseases^40^, and social status^41^. Second, our findings suggest that these anatomical measures can be used to identify an individual subject. Thus, these measures provide the potential for complementing those techniques currently used for subject identification (e.g., fingerprints, eye features). Although these “brain print” applications are potentially interesting, we would like to emphasize that we are more interested in the obvious “individuality” of the human brain.

For identification, we used two frequently applied statistical techniques – LDA and WKNN. LDA is a relatively simple and rapid operating mathematical procedure, while WKNN is a non-parametric exhaustive search procedure that requires a substantial amount of computational resources. As we have already mentioned in the method section, LDA and WKNN are both sensitive to distance measures representing the difference between the variables (here anatomical measures).

LDA is a parametric classifier using Fisher’s distance metric. Learning in this algorithm means finding the mean and covariance of the training dataset. For classification and identification, the classifier uses these means and covariances and determines the minimum distance between the subjects. At the end, LDA provides a linear function with weighted predictors (here anatomical measures), which is used for subject identification.

WKNN on the other hand is a nonparametric method, which uses the inverse squared Euclidian distance between each subject. A major disadvantage of this technique is that it is actually impossible to delineate which predictors contribute most effectively to the identification result. This, however, can be achieved by using the stepwise LDA as we have done in our study. Thus, by identifying the particular predictors (here anatomical measures) contributing to the identification result, it is possible to delineate those anatomical features which are highly individual and also stable across time.

### Limitations

There are some limitations to mention here. First of all, we only have anatomical data from three successive time points for our identification computations. This is the minimum number of time points for the necessary computations. It would be much better and more valid to work with more than three time points, since more time points are generally associated with increased variance. However, since anatomical measures change in older age approximately <  = 1% per year^17,18,42^, it is only meaningful to obtain the anatomical measures on a yearly basis. For this project, we used anatomical data obtained over a time period of two years during which the subjects were scanned on a yearly basis. However, we will continue with the measurements and obtain anatomical measures for the subjects for the next 2–3 years. With these data, we plan to validate our identification results. A second limitation pertains to the sample from which we have obtained the anatomical measures. Here, we have used subjects older than 65 years. Whether the identification results would have been different for younger subjects has to be shown in future projects. We anticipate even more stability over the time periods, and thus very good identification results also for younger subjects. Third, we have used FreeSurfer software to compute the anatomical measures. Thus, some as-yet-unknown procedural aspects (e.g., related to the usage of the FreeSurfer package) might have influenced the anatomical measurements. However, since the FreeSurfer procedures are identical for all subjects, we anticipate only negligible influences from the FreeSurfer method.

## Conclusion

With this paper, we demonstrate that only the combination of a relatively small number of neuroanatomical features can be used to identify individual subjects with relatively high precision. Even the easy-to-apply LDA technique provides very good identification results. Thus, this study demonstrates that human brain anatomy is highly individual.

## Electronic supplementary material

Supplementary information
