# Supplementary material for: Identification of individual subjects on the basis of their brain anatomical features
Source: Sci Rep. 2018 Apr 4;8:5611. doi: 10.1038/s41598-018-23696-6 (PMC5884835; doi:10.1038/s41598-018-23696-6)
Supplement: Supplementary file 1 — Supplementary information [file 41598_2018_23696_MOESM1_ESM.pdf]

	Left Hemisphere (Area Features)		Left Hemisphere (Thickness Features)		Left Hemisphere (Volume Features)		Right Hemisphere (Area Features)		Right Hemisphere (Thickness Features)		Right Hemisphere (Volume Features)		
1	lh_G_and_S_frontomargin_area	76	lh_G_and_S_frontomargin_thickness	151	lh_G_and_S_frontomargin_volume	225	rh_G_and_S_frontomargin_area	300	rh_G_and_S_frontomargin_thickness	375	rh_G_and_S_frontomargin_volume	449	Left-Lateral-Ventricle
2	lh_G_and_S_occipital_inf_area	77	lh_G_and_S_occipital_inf_thickness	152	lh_G_and_S_occipital_inf_volume	226	rh_G_and_S_occipital_inf_area	301	rh_G_and_S_occipital_inf_thickness	376	rh_G_and_S_occipital_inf_volume	450	Left-Inf-Lat-Vent
3	lh_G_and_S_paracentral_area	78	lh_G_and_S_paracentral_thickness	153	lh_G_and_S_paracentral_volume	227	rh_G_and_S_paracentral_area	302	rh_G_and_S_paracentral_thickness	377	rh_G_and_S_paracentral_volume	451	Left-Cerebellum-White-Matter
4	lh_G_and_S_subcentral_area	79	lh_G_and_S_subcentral_thickness	154	lh_G_and_S_subcentral_volume	228	rh_G_and_S_subcentral_area	303	rh_G_and_S_subcentral_thickness	378	rh_G_and_S_subcentral_volume	452	Left-Cerebellum-Cortex
5	lh_G_and_S_transv_frontopol_area	80	lh_G_and_S_transv_frontopol_thickness	155	lh_G_and_S_transv_frontopol_volume	229	rh_G_and_S_transv_frontopol_area	304	rh_G_and_S_transv_frontopol_thickness	379	rh_G_and_S_transv_frontopol_volume	453	Left-Thalamus-Proper
6	lh_G_and_S_cingul-Ant_area	81	lh_G_and_S_cingul-Ant_thickness	156	lh_G_and_S_cingul-Ant_volume	230	rh_G_and_S_cingul-Ant_area	305	rh_G_and_S_cingul-Ant_thickness	380	rh_G_and_S_cingul-Ant_volume	454	Left-Caudate
7	lh_G_and_S_cingul-Mid-Ant_area	82	lh_G_and_S_cingul-Mid-Ant_thickness	157	lh_G_and_S_cingul-Mid-Ant_volume	231	rh_G_and_S_cingul-Mid-Ant_area	306	rh_G_and_S_cingul-Mid-Ant_thickness	381	rh_G_and_S_cingul-Mid-Ant_volume	455	Left-Putamen
8	lh_G_and_S_cingul-Mid-Post_area	83	lh_G_and_S_cingul-Mid-Post_thickness	158	lh_G_and_S_cingul-Mid-Post_volume	232	rh_G_and_S_cingul-Mid-Post_area	307	rh_G_and_S_cingul-Mid-Post_thickness	382	rh_G_and_S_cingul-Mid-Post_volume	456	Left-Pallidum
9	lh_G_cingul-Post-dorsal_area	84	lh_G_cingul-Post-dorsal_thickness	159	lh_G_cingul-Post-dorsal_volume	233	rh_G_cingul-Post-dorsal_area	308	rh_G_cingul-Post-dorsal_thickness	383	rh_G_cingul-Post-dorsal_volume	457	3rd-Ventricle
10	lh_G_cingul-Post-ventral_area	85	lh_G_cingul-Post-ventral_thickness	160	lh_G_cingul-Post-ventral_volume	234	rh_G_cingul-Post-ventral_area	309	rh_G_cingul-Post-ventral_thickness	384	rh_G_cingul-Post-ventral_volume	458	4th-Ventricle
11	lh_G_cuneus_area	86	lh_G_cuneus_thickness	161	lh_G_cuneus_volume	235	rh_G_cuneus_area	310	rh_G_cuneus_thickness	385	rh_G_cuneus_volume	459	Brain-Stem
12	lh_G_front_inf-Opercular_area	87	lh_G_front_inf-Opercular_thickness	162	lh_G_front_inf-Opercular_volume	236	rh_G_front_inf-Opercular_area	311	rh_G_front_inf-Opercular_thickness	386	rh_G_front_inf-Opercular_volume	460	Left-Hippocampus
13	lh_G_front_inf-Orbital_area	88	lh_G_front_inf-Orbital_thickness	163	lh_G_front_inf-Orbital_volume	237	rh_G_front_inf-Orbital_area	312	rh_G_front_inf-Orbital_thickness	387	rh_G_front_inf-Orbital_volume	461	Left-Amygdala
14	lh_G_front_inf-Triangul_area	89	lh_G_front_inf-Triangul_thickness	164	lh_G_front_inf-Triangul_volume	238	rh_G_front_inf-Triangul_area	313	rh_G_front_inf-Triangul_thickness	388	rh_G_front_inf-Triangul_volume	462	CSF
15	lh_G_front_middle_area	90	lh_G_front_middle_thickness	165	lh_G_front_middle_volume	239	rh_G_front_middle_area	314	rh_G_front_middle_thickness	389	rh_G_front_middle_volume	463	Left-Accumbens-area
16	lh_G_front_sup_area	91	lh_G_front_sup_thickness	166	lh_G_front_sup_volume	240	rh_G_front_sup_area	315	rh_G_front_sup_thickness	390	rh_G_front_sup_volume	464	Left-VentralDC
17	lh_G_Ins_lg_and_S_cent_ins_area	92	lh_G_Ins_lg_and_S_cent_ins_thickness	167	lh_G_Ins_lg_and_S_cent_ins_volume	241	rh_G_Ins_lg_and_S_cent_ins_area	316	rh_G_Ins_lg_and_S_cent_ins_thickness	391	rh_G_Ins_lg_and_S_cent_ins_volume	465	Left-vessel
18	lh_G_insular_short_area	93	lh_G_insular_short_thickness	168	lh_G_insular_short_volume	242	rh_G_insular_short_area	317	rh_G_insular_short_thickness	392	rh_G_insular_short_volume	466	Left-choroid-plexus
19	lh_G_occipital_middle_area	94	lh_G_occipital_middle_thickness	169	lh_G_occipital_middle_volume	243	rh_G_occipital_middle_area	318	rh_G_occipital_middle_thickness	393	rh_G_occipital_middle_volume	467	Right-Lateral-Ventricle
20	lh_G_occipital_sup_area	95	lh_G_occipital_sup_thickness	170	lh_G_occipital_sup_volume	244	rh_G_occipital_sup_area	319	rh_G_occipital_sup_thickness	394	rh_G_occipital_sup_volume	468	Right-Inf-Lat-Vent
21	lh_G_oc-temp_lat-fusifor_area	96	lh_G_oc-temp_lat-fusifor_thickness	171	lh_G_oc-temp_lat-fusifor_volume	245	rh_G_oc-temp_lat-fusifor_area	320	rh_G_oc-temp_lat-fusifor_thickness	395	rh_G_oc-temp_lat-fusifor_volume	469	Right-Cerebellum-White-Matter
22	lh_G_oc-temp_med-Lingual_area	97	lh_G_oc-temp_med-Lingual_thickness	172	lh_G_oc-temp_med-Lingual_volume	246	rh_G_oc-temp_med-Lingual_area	321	rh_G_oc-temp_med-Lingual_thickness	396	rh_G_oc-temp_med-Lingual_volume	470	Right-Cerebellum-Cortex
23	lh_G_oc-temp_med-Parahip_area	98	lh_G_oc-temp_med-Parahip_thickness	173	lh_G_oc-temp_med-Parahip_volume	247	rh_G_oc-temp_med-Parahip_area	322	rh_G_oc-temp_med-Parahip_thickness	397	rh_G_oc-temp_med-Parahip_volume	471	Right-Thalamus-Proper
24	lh_G_orbital_area	99	lh_G_orbital_thickness	174	lh_G_orbital_volume	248	rh_G_orbital_area	323	rh_G_orbital_thickness	398	rh_G_orbital_volume	472	Right-Caudate
25	lh_G_pariet_inf-Angular_area	100	lh_G_pariet_inf-Angular_thickness	175	lh_G_pariet_inf-Angular_volume	249	rh_G_pariet_inf-Angular_area	324	rh_G_pariet_inf-Angular_thickness	399	rh_G_pariet_inf-Angular_volume	473	Right-Putamen
26	lh_G_pariet_inf-Supramar_area	101	lh_G_pariet_inf-Supramar_thickness	176	lh_G_pariet_inf-Supramar_volume	250	rh_G_pariet_inf-Supramar_area	325	rh_G_pariet_inf-Supramar_thickness	400	rh_G_pariet_inf-Supramar_volume	474	Right-Pallidum
27	lh_G_parietal_sup_area	102	lh_G_parietal_sup_thickness	177	lh_G_parietal_sup_volume	251	rh_G_parietal_sup_area	326	rh_G_parietal_sup_thickness	401	rh_G_parietal_sup_volume	475	Right-Hippocampus
28	lh_G_postcentral_area	103	lh_G_postcentral_thickness	178	lh_G_postcentral_volume	252	rh_G_postcentral_area	327	rh_G_postcentral_thickness	402	rh_G_postcentral_volume	476	Right-Amygdala
29	lh_G_precentral_area	104	lh_G_precentral_thickness	179	lh_G_precentral_volume	253	rh_G_precentral_area	328	rh_G_precentral_thickness	403	rh_G_precentral_volume	477	Right-Accumbens-area
30	lh_G_precuneus_area	105	lh_G_precuneus_thickness	180	lh_G_precuneus_volume	254	rh_G_precuneus_area	329	rh_G_precuneus_thickness	404	rh_G_precuneus_volume	478	Right-VentralDC
31	lh_G_rectus_area	106	lh_G_rectus_thickness	181	lh_G_rectus_volume	255	rh_G_rectus_area	330	rh_G_rectus_thickness	405	rh_G_rectus_volume	479	Right-vessel
32	lh_G_subcallosal_area	107	lh_G_subcallosal_thickness	182	lh_G_subcallosal_volume	256	rh_G_subcallosal_area	331	rh_G_subcallosal_thickness	406	rh_G_subcallosal_volume	480	Right-choroid-plexus
33	lh_G_temp_sup-G_T_transv_area	108	lh_G_temp_sup-G_T_transv_thickness	183	lh_G_temp_sup-G_T_transv_volume	257	rh_G_temp_sup-G_T_transv_area	332	rh_G_temp_sup-G_T_transv_thickness	407	rh_G_temp_sup-G_T_transv_volume	481	5th-Ventricle
34	lh_G_temp_sup-Lateral_area	109	lh_G_temp_sup-Lateral_thickness	184	lh_G_temp_sup-Lateral_volume	258	rh_G_temp_sup-Lateral_area	333	rh_G_temp_sup-Lateral_thickness	408	rh_G_temp_sup-Lateral_volume	482	WM-hypointensities
35	lh_G_temp_sup-Plan_polar_area	110	lh_G_temp_sup-Plan_polar_thickness	185	lh_G_temp_sup-Plan_polar_volume	259	rh_G_temp_sup-Plan_polar_area	334	rh_G_temp_sup-Plan_polar_thickness	409	rh_G_temp_sup-Plan_polar_volume	483	Left-WM-hypointensities
36	lh_G_temp_sup-Plan_tempo_area	111	lh_G_temp_sup-Plan_tempo_thickness	186	lh_G_temp_sup-Plan_tempo_volume	260	rh_G_temp_sup-Plan_tempo_area	335	rh_G_temp_sup-Plan_tempo_thickness	410	rh_G_temp_sup-Plan_tempo_volume	484	Right-WM-hypointensities
37	lh_G_temporal_inf_area	112	lh_G_temporal_inf_thickness	187	lh_G_temporal_inf_volume	261	rh_G_temporal_inf_area	336	rh_G_temporal_inf_thickness	411	rh_G_temporal_inf_volume	485	non-WM-hypointensities
38	lh_G_temporal_middle_area	113	lh_G_temporal_middle_thickness	188	lh_G_temporal_middle_volume	262	rh_G_temporal_middle_area	337	rh_G_temporal_middle_thickness	412	rh_G_temporal_middle_volume	486	Left-non-WM-hypointensities
39	lh_Lat_Fis-ant-Horizont_area	114	lh_Lat_Fis-ant-Horizont_thickness	189	lh_Lat_Fis-ant-Horizont_volume	263	rh_Lat_Fis-ant-Horizont_area	338	rh_Lat_Fis-ant-Horizont_thickness	413	rh_Lat_Fis-ant-Horizont_volume	487	Right-non-WM-hypointensities
40	lh_Lat_Fis-ant-Vertical_area	115	lh_Lat_Fis-ant-Vertical_thickness	190	lh_Lat_Fis-ant-Vertical_volume	264	rh_Lat_Fis-ant-Vertical_area	339	rh_Lat_Fis-ant-Vertical_thickness	414	rh_Lat_Fis-ant-Vertical_volume	488	Optic-Chiasm
41	lh_Lat_Fis-post_area	116	lh_Lat_Fis-post_thickness	191	lh_Lat_Fis-post_volume	265	rh_Lat_Fis-post_area	340	rh_Lat_Fis-post_thickness	415	rh_Lat_Fis-post_volume	489	CC_Posterior
42	lh_Pole_occipital_area	117	lh_Pole_occipital_thickness	192	lh_Pole_occipital_volume	266	rh_Pole_occipital_area	341	rh_Pole_occipital_thickness	416	rh_Pole_occipital_volume	490	CC_Mid_Posterior
43	lh_Pole_temporal_area	118	lh_Pole_temporal_thickness	193	lh_Pole_temporal_volume	267	rh_Pole_temporal_area	342	rh_Pole_temporal_thickness	417	rh_Pole_temporal_volume	491	CC_Central
44	lh_S_calcarine_area	119	lh_S_calcarine_thickness	194	lh_S_calcarine_volume	268	rh_S_calcarine_area	343	rh_S_calcarine_thickness	418	rh_S_calcarine_volume	492	CC_Mid_Anterior
45	lh_S_central_area	120	lh_S_central_thickness	195	lh_S_central_volume	269	rh_S_central_area	344	rh_S_central_thickness	419	rh_S_central_volume	493	CC_Anterior
46	lh_S_cingul-Marginalis_area	121	lh_S_cingul-Marginalis_thickness	196	lh_S_cingul-Marginalis_volume	270	rh_S_cingul-Marginalis_area	345	rh_S_cingul-Marginalis_thickness	420	rh_S_cingul-Marginalis_volume	494	BrainSegVol
47	lh_S_circular_insula_ant_area	122	lh_S_circular_insula_ant_thickness	197	lh_S_circular_insula_ant_volume	271	rh_S_circular_insula_ant_area	346	rh_S_circular_insula_ant_thickness	421	rh_S_circular_insula_ant_volume	495	BrainSegVolNotVent
48	lh_S_circular_insula_inf_area	123	lh_S_circular_insula_inf_thickness	198	lh_S_circular_insula_inf_volume	272	rh_S_circular_insula_inf_area	347	rh_S_circular_insula_inf_thickness	422	rh_S_circular_insula_inf_volume	496	BrainSegVolNotVentSurf
49	lh_S_circular_insula_sup_area	124	lh_S_circular_insula_sup_thickness	199	lh_S_circular_insula_sup_volume	273	rh_S_circular_insula_sup_area	348	rh_S_circular_insula_sup_thickness	423	rh_S_circular_insula_sup_volume	497	lhCortexVol
50	lh_S_collat_transv_ant_area	125	lh_S_collat_transv_ant_thickness	200	lh_S_collat_transv_ant_volume	274	rh_S_collat_transv_ant_area	349	rh_S_collat_transv_ant_thickness	424	rh_S_collat_transv_ant_volume	498	rhCortexVol
51	lh_S_collat_transv_post_area	126	lh_S_collat_transv_post_thickness	201	lh_S_collat_transv_post_volume	275	rh_S_collat_transv_post_area	350	rh_S_collat_transv_post_thickness	425	rh_S_collat_transv_post_volume	499	CortexVol
52	lh_S_front_inf_area	127	lh_S_front_inf_thickness	202	lh_S_front_inf_volume	276	rh_S_front_inf_area	351	rh_S_front_inf_thickness	426	rh_S_front_inf_volume	500	lhCorticalWhiteMatterVol
53	lh_S_front_middle_area	128	lh_S_front_middle_thickness	203	lh_S_front_middle_volume	277	rh_S_front_middle_area	352	rh_S_front_middle_thickness	427	rh_S_front_middle_volume	501	rhCorticalWhiteMatterVol
54	lh_S_front_sup_area	129	lh_S_front_sup_thickness	204	lh_S_front_sup_volume	278	rh_S_front_sup_area	353	rh_S_front_sup_thickness	428	rh_S_front_sup_volume	502	CorticalWhiteMatterVol
55	lh_S_interm_prim-Jensen_area	130	lh_S_interm_prim-Jensen_thickness	205	lh_S_interm_prim-Jensen_volume	279	rh_S_interm_prim-Jensen_area	354	rh_S_interm_prim-Jensen_thickness	429	rh_S_interm_prim-Jensen_volume	503	SubCortGrayVol
56	lh_S_intrapariet_and_P_trans_area	131	lh_S_intrapariet_and_P_trans_thickness	206	lh_S_intrapariet_and_P_trans_volume	280	rh_S_intrapariet_and_P_trans_area	355	rh_S_intrapariet_and_P_trans_thickness	430	rh_S_intrapariet_and_P_trans_volume	504	TotalGrayVol
57	lh_S_oc_middle_and_Lunatus_area	132	lh_S_oc_middle_and_Lunatus_thickness	207	lh_S_oc_middle_and_Lunatus_volume	281	rh_S_oc_middle_and_Lunatus_area	356	rh_S_oc_middle_and_Lunatus_thickness	431	rh_S_oc_middle_and_Lunatus_volume	505	SupraTentorialVol
58	lh_S_oc_sup_and_transversal_area	133	lh_S_oc_sup_and_transversal_thickness	208	lh_S_oc_sup_and_transversal_volume	282	rh_S_oc_sup_and_transversal_area	357	rh_S_oc_sup_and_transversal_thickness	432	rh_S_oc_sup_and_transversal_volume	506	SupraTentorialVolNotVent
59	lh_S_occipital_ant_area	134	lh_S_occipital_ant_thickness	209	lh_S_occipital_ant_volume	283	rh_S_occipital_ant_area	358	rh_S_occipital_ant_thickness	433	rh_S_occipital_ant_volume	507	SupraTentorialVolNotVentVox
60	lh_S_oc-temp_lat_area	135	lh_S_oc-temp_lat_thickness	210	lh_S_oc-temp_lat_volume	284	rh_S_oc-temp_lat_area	359	rh_S_oc-temp_lat_thickness	434	rh_S_oc-temp_lat_volume	508	MaskVol
61	lh_S_oc-temp_med_and_Lingual_area	136	lh_S_oc-temp_med_and_Lingual_thickness	211	lh_S_oc-temp_med_and_Lingual_volume	285	rh_S_oc-temp_med_and_Lingual_area	360	rh_S_oc-temp_med_and_Lingual_thickness	435	rh_S_oc-temp_med_and_Lingual_volume	509	BrainSegVol-to-eTIV
62	lh_S_orbital_lateral_area	137	lh_S_orbital_lateral_thickness	212	lh_S_orbital_lateral_volume	286	rh_S_orbital_lateral_area	361	rh_S_orbital_lateral_thickness	436	rh_S_orbital_lateral_volume	510	MaskVol-to-eTIV
63	lh_S_orbital_med-olfact_area	138	lh_S_orbital_med-olfact_thickness	213	lh_S_orbital_med-olfact_volume	287	rh_S_orbital_med-olfact_area	362	rh_S_orbital_med-olfact_thickness	437	rh_S_orbital_med-olfact_volume	511	lhSurfaceHoles
64	lh_S_orbital-H_Shaped_area	139	lh_S_orbital-H_Shaped_thickness	214	lh_S_orbital-H_Shaped_volume	288	rh_S_orbital-H_Shaped_area	363	rh_S_orbital-H_Shaped_thickness	438	rh_S_orbital-H_Shaped_volume	512	rhSurfaceHoles
65	lh_S_parieto_occipital_area	140	lh_S_parieto_occipital_thickness	215	lh_S_parieto_occipital_volume	289	rh_S_parieto_occipital_area	364	rh_S_parieto_occipital_thickness	439	rh_S_parieto_occipital_volume	513	SurfaceHoles
66	lh_S_pericallosal_area	141	lh_S_pericallosal_thickness	216	lh_S_pericallosal_volume	290	rh_S_pericallosal_area	365	rh_S_pericallosal_thickness	440	rh_S_pericallosal_volume	514	EstimatedTotalIntraCranialVol
67	lh_S_postcentral_area	142	lh_S_postcentral_thickness	217	lh_S_postcentral_volume	291	rh_S_postcentral_area	366	rh_S_postcentral_thickness	441	rh_S_postcentral_volume		
68	lh_S_precentral-inf-part_area	143	lh_S_precentral-inf-part_thickness	218	lh_S_precentral-inf-part_volume	292	rh_S_precentral-inf-part_area	367	rh_S_precentral-inf-part_thickness	442	rh_S_precentral-inf-part_volume		
69	lh_S_precentral-sup-part_area	144	lh_S_precentral-sup-part_thickness	219	lh_S_precentral-sup-part_volume	293	rh_S_precentral-sup-part_area	368	rh_S_precentral-sup-part_thickness	443	rh_S_precentral-sup-part_volume		
70	lh_S_suborbital_area	145	lh_S_suborbital_thickness	220	lh_S_suborbital_volume	294	rh_S_suborbital_area	369	rh_S_suborbital_thickness	444	rh_S_suborbital_volume		
71	lh_S_subparietal_area	146	lh_S_subparietal_thickness	221	lh_S_subparietal_volume	295	rh_S_subparietal_area	370	rh_S_subparietal_thickness	445	rh_S_subparietal_volume		
72	lh_S_temporal_inf_area	147	lh_S_temporal_inf_thickness	222	lh_S_temporal_inf_volume	296	rh_S_temporal_inf_area	371	rh_S_temporal_inf_thickness	446	rh_S_temporal_inf_volume		
73	lh_S_temporal_sup_area	148	lh_S_temporal_sup_thickness	223	lh_S_temporal_sup_volume	297	rh_S_temporal_sup_area	372	rh_S_temporal_sup_thickness	447	rh_S_temporal_sup_volume		
74	lh_S_temporal_transverse_area	149	lh_S_temporal_transverse_thickness	224	lh_S_temporal_transverse_volume	298	rh_S_temporal_transverse_area	373	rh_S_temporal_transverse_thickness	448	rh_S_temporal_transverse_volume		
75	lh_WhiteSurfArea_area	150	lh_MeanThickness_thickness	*	total LH Volume	299	rh_WhiteSurfArea_area	374	rh_MeanThickness_thickness	*	total RH Volume		

Supplementary document for anatomical study

Table 2: Summary description of the used anatomical datasets. Indicated are the acronyms of the datasets along with the number of anatomical measures included.

No	Dataset	Scaled
1	11 LBR	11
2	THICKNESS	148
3	AREA	148
4	VOLUME	148
5	ALL	510

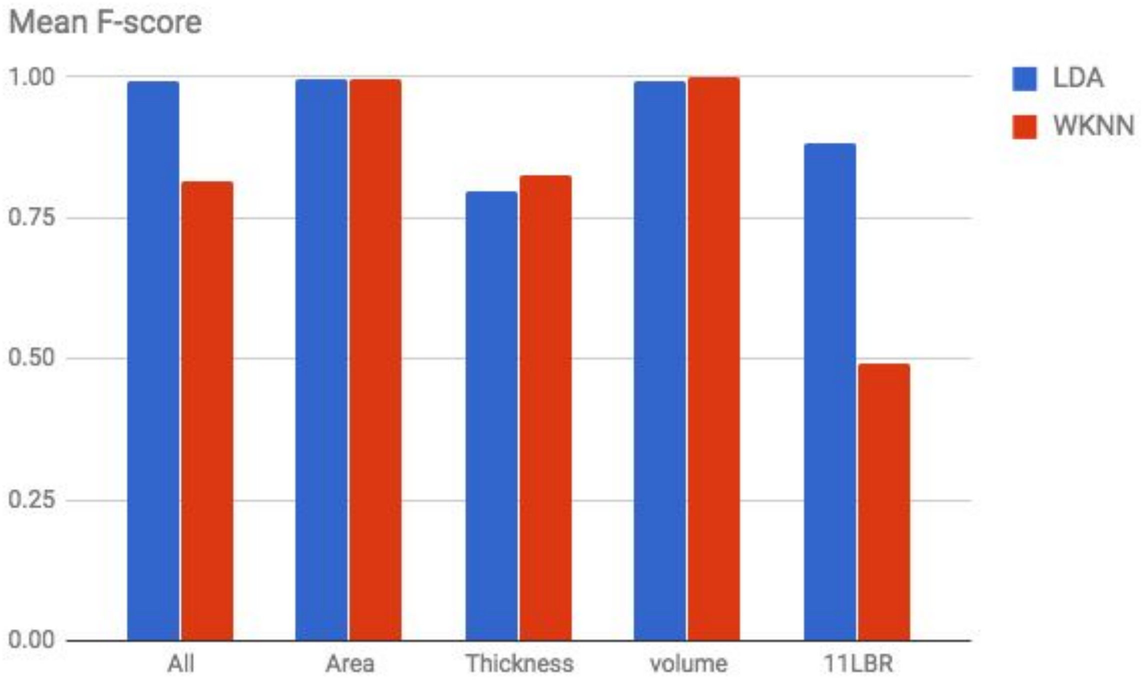

Figure 1: Mean F-score across the different noise levels broken down for the different identification techniques. Please note, the larger the F-score the better is the identification result.

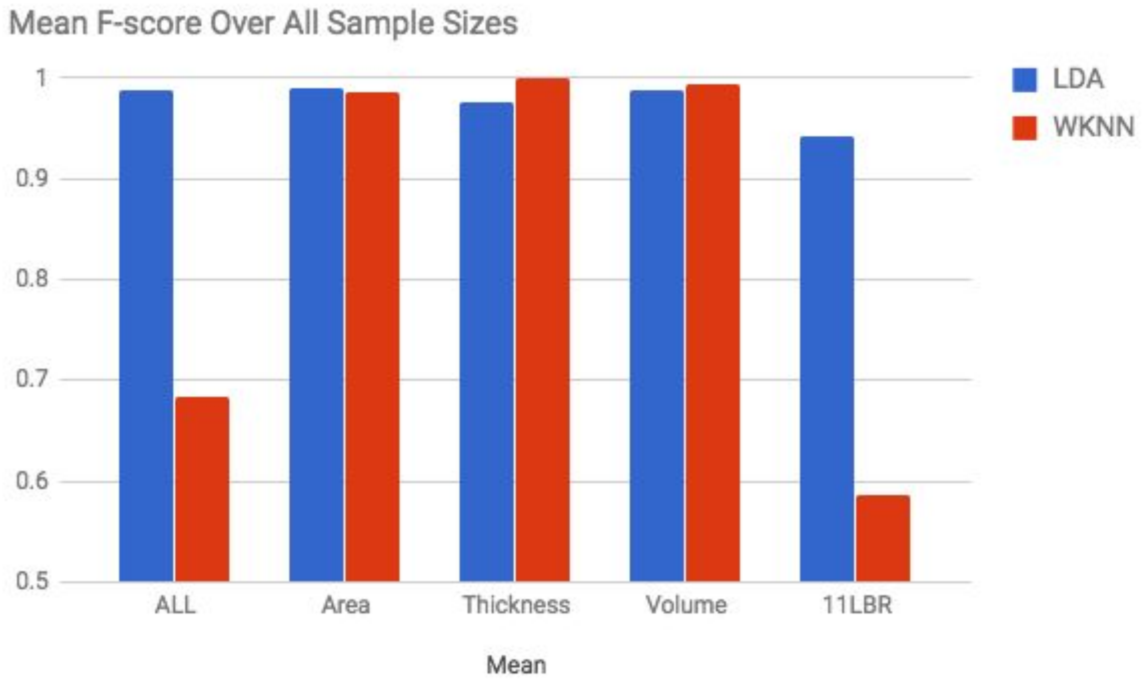

Figure 2: Mean F-scores across the different sample sizes broken down for the different identification techniques.

Table 2: Sample confusion matrix

<table>
<tr><th>Number</th><th colspan="2">confusionmatrix</th><th>Acc</th><th>Sens</th><th>Spec</th><th>F1</th></tr>
<tr><td rowspan="2">1</td><td>1</td><td>0</td><td rowspan="2">1.00</td><td rowspan="2">1.00</td><td rowspan="2">1.00</td><td rowspan="2">1.00</td></tr>
<tr><td>0</td><td>190</td></tr>
<tr><td rowspan="2">2</td><td>0</td><td>0</td><td rowspan="2">1.00</td><td rowspan="2">0</td><td rowspan="2">1.00</td><td rowspan="2">NAN=eps</td></tr>
<tr><td>1</td><td>19</td></tr>
</table>
